# To B or Not to B: Mechanisms of Protection Conferred by rVSV-EBOV-GP and the Roles of Innate and Adaptive Immunity

**DOI:** 10.3390/microorganisms8101473

**Published:** 2020-09-25

**Authors:** Amanda N. Pinski, Ilhem Messaoudi

**Affiliations:** 1 Department of Molecular Biology and Biochemistry, University of California, Irvine, Irvine, CA 92697, USA; apinski@uci.edu; 2 Center for Virus Research, University of California, Irvine, Irvine, CA 92697, USA; 3 Institute for Immunology, University of California, Irvine, Irvine, CA 92697, USA

**Keywords:** Ebola virus, EBOV, rVSV-EBOV-GP, vaccine, protection, efficacy, nonhuman primates

## Abstract

Zaire Ebola virus (EBOV) is a member of the *Filoviridae* family of negative sense, single-stranded RNA viruses. EBOV infection causes Ebola virus disease (EVD), characterized by coagulopathy, lymphopenia, and multi-organ failure, which can culminate in death. In 2019, the FDA approved the first vaccine against EBOV, a recombinant live-attenuated viral vector wherein the G protein of vesicular stomatitis virus is replaced with the glycoprotein (GP) of EBOV (rVSV-EBOV-GP, Ervebo^®^ by Merck). This vaccine demonstrates high efficacy in nonhuman primates by providing prophylactic, rapid, and post-exposure protection. In humans, rVSV-EBOV-GP demonstrated 100% protection in several phase III clinical trials in over 10,000 individuals during the 2013–2016 West Africa epidemic. As of 2020, over 218,000 doses of rVSV-EBOV-GP have been administered to individuals with high risk of EBOV exposure. Despite licensure and robust preclinical studies, the mechanisms of rVSV-EBOV-GP-mediated protection are not fully understood. Such knowledge is crucial for understanding vaccine-mediated correlates of protection from EVD and to aid the further design and development of therapeutics against filoviruses. Here, we summarize the current literature regarding the host response to vaccination and EBOV exposure, and evidence regarding innate and adaptive immune mechanisms involved in rVSV-EBOV-GP-mediated protection, with a focus on the host transcriptional response. Current data strongly suggest a protective synergy between rapid innate and humoral immunity.

## 1. Introduction

Ebola virus, a member of the negative-sense, single stranded RNA virus family *Filoviridae*, is the causative agent of Ebola virus disease (EVD) [1,2]. EVD is characterized by hemorrhaging, dysregulated immune activation, excessive inflammation and aberrant cytokine secretion that ultimately lead massive organ failure and death. Case fatality rates (CFR) range from 25% to 90% depending on the species [3,4,5,6,7,8,9,10] (Table 1). Six species of Ebola virus have been identified, including Zaire (EBOV, previously known as ZEBOV), Sudan (SUDV), Tai Forest (TAFV), Bundibugyo (BDBV), Reston (RESTV), and Bombali (BOMV) Ebola viruses. Only the first four species are known to cause EVD in both humans and nonhuman primates while Reston and Bombali species have no reported cases in humans to date [11,12].

Ebola virus was first discovered in 1976 in Zaire, which is now the Democratic Republic of Congo (DRC) [13]. This outbreak of EBOV lead to over 300 cases with a CFR of 88% [13]. Simultaneously, an outbreak of 280 cases (CFR = 53%) in Sudan caused by SUDV occurred [13]. Subsequent outbreaks led to the discovery of four additional strains of Ebola virus (Table 1). An outbreak of hemorrhagic fever among cynomolgus macaques in the United States in 1989 led to the identification of RESTV, which is not pathogenic to humans despite seroconversion in high-risk exposures [12,14,15,16]. A single nonfatal case of TAFV was identified in 1994 in an ethologist who conducted a necropsy of a nonhuman primate but no cases have been reported since [17]. BDBV was discovered in Uganda in 2007, where it caused 149 cases with a CFR of 25%, notably lower than that of EBOV and SUDV [18,19,20]. Lastly, BOMV was recently discovered in free-tailed bats in Sierra Leone, and, although reported to bind and mediate entry into human cells, its pathogenicity humans is currently undefined [21].

Most Ebola virus outbreaks since 1976 have been caused by various strains of the EBOV species. The 1976 EBOV outbreak of 318 cases was caused by the Mayinga strain (CFR = 88%) while a subsequent outbreak of 315 cases in 1995 (CFR = 79%) was caused by the Kikwit strain, which shares over 97% sequence similarity and incurs similar CFR [22,23,24,25,26,27]. The 2013-2016 West Africa outbreak which culminated in over 28,000 cases and 11,308 deaths was caused by the EBOV Makona strain, which despite sharing 97% sequence similarity with Mayinga and Kikwit, has a notably lower CFR of ~40% [28,29]. This epidemic (10 countries were involved) demonstrated the capacity for vast Ebola virus transmission and instituted a state of emergency that facilitated the development of prophylactics and therapeutics. Furthermore, recent and ongoing EBOV outbreaks in the Democratic Republic of Congo (DRC) at the time of this writing, including the EBOV Ituri outbreak affecting over 3,000 individuals (CFR = 66%), emphasize the need for continued developments of therapeutics and vaccines [30]. The rVSV-EBOV-GP vaccine, which demonstrated great efficacy in nonhuman primates (NHPs) and humans [31,32,33,34,35,36], was approved in December 2019 after several phase III clinical and ring-vaccination trials demonstrated 100% protection in the absence of severe adverse events in over 10,000 individuals [37,38,39,40,41,42]. Additionally, over 200,000 doses were administered as part of compassionate use prior to FDA approval due to high efficacy and the 2018 outbreak in the DRC and Uganda (Table 1) [43].

Despite these advances, the mechanisms of rVSV-EBOV-induced protection remain unclear. Data from NHP model studies indicate a critical role for humoral immunity and a marginal contribution of CD8 T cell responses in mediating protection when the vaccine is administered at least 7 days before challenge. On the other hand, rapid protection (when the vaccine is administered <7 days before challenge) indicate a critical role for early induction of innate antiviral responses leading to effective humoral responses. In this review, we summarize the current knowledge regarding the correlates and mechanisms of rVSV-EBOV-mediated protection.

## 2. rVSV-EBOV-GP Vaccinology

rVSV-EBOV-GP is a live, recombinant vaccine constructed from the vesicular stomatitis virus (VSV) vector platform [36]. Although capable of infecting other animals including horses, cattle and swine, VSV rarely infects humans, who are largely asymptomatic or exhibit only mild cases of disease following infection [44,45]. The use of this vector avoids potential issues surrounding pre-existing immunity encountered with some of the other vaccine vectors [44,45]. The attenuation of VSV is achieved by replacing the native VSV glycoprotein (G) with the glycoprotein of a different virus [46,47,48,49]. The ease of genetic manipulation, robust gene expression and a lack of wide-spread pre-existing immunity make this virus an ideal vector [50]. In addition to Ebola virus, the VSV platform has also been assessed for protection against other pathogenic viruses, including influenza virus, severe acute respiratory syndrome (SARS) coronavirus (SARS-CoV-2), and human immunodeficiency virus (HIV), demonstrating high levels of immunogenicity and 100% protection against viral challenge [51,52,53,54,55]. However, none are currently licensed.

The Ebola virus glycoprotein (GP) included in the FDA-approved rVSV-EBOV-GP vaccine (Merck™, Kenilworth, NJ, USA) used in preclinical and clinical studies is derived from the Kikwit strain [37,38,46,56]. GP from the Makona and Mayinga strains have also been successfully evaluated in NHP [57,58]. High sequence similarity between all strains of EBOV ensure intra-species protection but not necessarily inter-species protection [56,59,60,61,62]. For instance, vaccination with rVSV vector expressing the EBOV GP or SUDV GP does not fully protect nonhuman primates challenged with a lethal dose of BDBV [62]. Moreover, rVSV-TAFV-GP does not protect NHPs from lethal challenge with BDBV, while rVSV-EBOV-GP offers partial protection (75%). Future applications may utilize the GP of different Ebola virus species, although current efforts focus on protection against EBOV since it is the cause of multiple past and ongoing outbreaks [28,29] (Table 1).

## 3. Preclinical Studies in Nonhuman Primates

Initial studies assessed immunogenicity and efficacy of rVSV-EBOV-GP in mice and small animal models [36,46,63,64,65,66,67]. However, rodent models do not fully recapitulate human EVD disease and require mouse-adapted strains [68,69]. Mutations in mouse-adapted EBOV affect key viral proteins involved in the viral life cycle and immune antagonism of human innate immune response [70,71]. Mice infected with wild-type EBOV exhibit no clinical pathology while those challenged with mouse-adapted EBOV recapitulate EBOV-induced lymphopenia and inflammation but not coagulopathy [70,71,72]. Safety and efficacy of rVSV-EBOV-GP was subsequently demonstrated in NHPs [35,36,46,57,61,66,67,73], particularly cynomolgus and rhesus macaques, which are considered the gold standard animal model since they exhibit the hemorrhaging, coagulopathy, lymphopenia, and the cytokine storm characteristic of EVD [74,75,76,77].

A single dose of rVSV-EBOV-GP (10^7^ plaque forming units, PFU) fully protects (absence of viremia and disease) cynomolgus macaques vaccinated via intramuscular, intranasal, or oral routes when it is administered 28 days before challenge with a lethal dose of EBOV Kikwit, EBOV Mayinga or EBOV Makona [35,36,56,57,73]. A more recent study showed that a dose as little as 10 PFU of rVSV-EBOV-GP, which is one millionth of the standard dose for NHPs (10^7^ PFU) and nearly 10 millionth of the dose given to humans (7.2 × 10^7^ PFU), fully protected NHPs from lethal EBOV Makona challenge, albeit with mild viremia and clinical symptoms [57]. The full protection afforded by rVSV-EBOV-GP also extends to NHPs vaccinated as little as 7 days before challenge with EBOV Makona [56]. Furthermore, a portion of NHPs vaccinated 1–3 days before challenge (67%) or up to 24 h after challenge (33–67%) with EBOV Makona, and 20–30 min post-exposure to EBOV Kikwit (50%), survive challenge [58,78,79,80]. The lack of neurovirulence in NHPs and the absence of adverse side effects and partial protection in immunocompromised macaques (infected with simian human immunodeficiency virus, SHIV) further demonstrate the safety of rVSV-EBOV-GP [35,81]. Collectively, these observations suggest the utility of rVSV-EBOV-GP as both a safe and efficacious prophylactic and post-exposure treatment for Ebola virus infection [59].

## 4. Clinical Studies to Market

The nearly two dozen human clinical trials conducted since the beginning of the 2013–2016 West Africa outbreak accelerated the development of rVSV-EBOV-GP to market (Table 2) [64]. Initial phase I and II clinical trials focused on dose escalation and safety [82,83,84,85,86,87,88,89,90]. These initial studies demonstrated that, at all doses tested (300,000 PFU-50 million PFU), patients developed EBOV-GP-specific antibodies and were defined as seropositive within 14 to 28 days post vaccination (DPV) [82,83,84,85,86,87,88,89,90]. However, the magnitudes of IgG titers were dose-dependent and higher doses were associated with a more diverse antibody repertoire following vaccination [89]. Neutralizing antibodies were seen as early as 28 DPV and were dominated by EBOV-GP-specific IgM at all doses [89]. Furthermore, the persistence of seropositivity at two years post-vaccination was 100% in subjects receiving at least 10 million PFU and 89% of those receiving 300,000 PFU of rVSV-EBOV-GP [82,84,85]. Multiple dosing schemes were not found to be superior since no significant increases in IgG or neutralizing antibody titers were detected after additional vaccinations [89]. Thus, coupled with the fact that rVSV-EBOV-GP is licensed for single-dosing, this review focuses on single-dose vaccination with rVSV-EBOV-GP.

rVSV-EBOV-GP is safe in children (6–12 years) (PACTR201411000919191, NCT03031912), adolescents (13–17 years), and immunocompromised individuals (HIV positive) (NCT03031912), although IgG levels were found to be notably lower in the latter group in line with previous observations in SHIV-infected NHPs [82,88,91]. Transient side effects from rVSV-EBOV-GP were frequent in all clinical trials and predominantly included headache, fatigue, muscle pain, arthralgia and fever. These symptoms frequently resolved within a week of vaccination [39,40,41,42,82,83,84,85,86,87,88,89,90,92]. Severe adverse side effects were rare and often correlated with pre-existing conditions.

Three phase III clinical trials conducted during 2015–2016 in populations of low- and high-risk to EBOV exposure ultimately demonstrated the efficacy of rVSV-EBOV-GP in both children and adults [39,40,41,42]. The first phase III clinical trial (PACTR2015030011057193) was an open-label, cluster-randomized ring vaccination trial occurring in Guinea April–July 2015 [40,41]. Individuals with laboratory-confirmed EVD and their contacts aged 18 years and older were grouped into clusters that were then randomly assigned to two groups: immediate (4123 people) or 21-day delayed (3528 people) vaccination with 2 × 10^7^ PFU of rVSV-EBOV. Index cases in both groups were similar in terms of age, gender and days post symptom onset to case reporting. Contacts in both groups were also similar with regards to number of contacts per cluster and age, although immediate vaccination clusters tended to have more high-risk contacts. Vaccine efficacy, evaluated as the number of EVD cases at 10 days post randomization, was 100% in the immediate vaccination group, which reported zero cases in comparison to 16 cases reported in the delayed vaccination group. Overall vaccine efficacy was 75.1% when considering all clusters. Given the high efficacy, delayed vaccination was discontinued and immediate vaccination was offered to additional individuals including children aged 6-18 years (*n* = 194) and adults (*n* = 5643) [39]. No cases of EVD were reported in either adults or children during the duration of the trial.

The second phase 2/3 clinical trial in Sierra Leone (Sierra Leone Trial to Induce a Vaccine against Ebola; STRIVE) (NCT02378753, PACTR201502001027220) occurred later in 2015 and ended in late December 2015 [42,93,94]. This open-label individually randomized controlled phase 2/3 clinical trial in Sierra Leone enrolled over 8000 healthcare and frontline workers to study the immunogenicity, efficacy and safety of rVSV-EBOV. These individuals were considered to have a 100-fold greater risk for Ebola exposure and EVD compared to the general population based on a previous study that compared infection rate in the general population and healthcare workers (≥15 years) in Sierra Leone [95]. Like the first phase III clinical trial, participants were randomly assigned to either immediate vaccination or delayed vaccination. No EVD cases or vaccine-related severe adverse events were reported in either group, again demonstrating excellent efficacy in high-risk settings. Additionally, rVSV-EBOV-GP was shown to be safe in early pregnancy.

A final phase III, randomized double-blind, multi-center clinical trial in Canada, Spain and the US (NCT02503202) examined the safety of two doses of rVSV-EBOV-GP(2 × 10^7^ or 10 × 10^7^ PFU) and durability of EBOV-GP-specific IgG in 1196 healthy adults with low risk of exposure to EBOV [39,96]. As described in other clinical trials, transient adverse events, such as arthralgia, were common in low and high doses of rVSV-EBOV. Approximately 94% of all individuals developed EBOV-GP-specific antibodies that persisted at the 24-month follow-up. The results of these successful clinical trials, coupled with the ongoing Ebola virus outbreak in the DRC and Uganda since 2018, resulted in the additional distribution of over 200,000 doses as compassionate use [30,43]. The rVSV-EBOV-GP vaccine (Ervebo™, Merck) was later approved by the FDA in December 2019 [29,37,38].

## 5. Host Response to rVSV-EBOV-GP Vaccination

**Figure 1:**
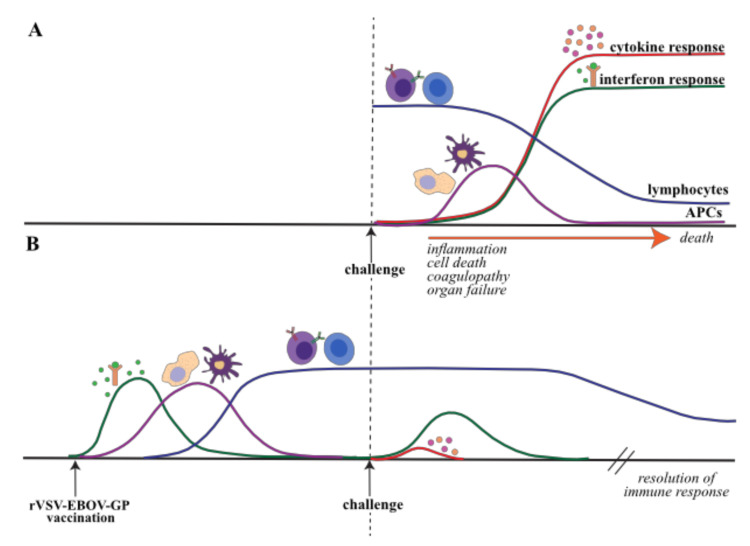
Host response to EBOV infection and rVSV-EBOV-GP vaccination. (**A**) EBOV infection induces a strong, sustained secretion of cytokines and interferon by myeloid cells. Impaired activation of APCs coupled with apoptosis induced by pro inflammatory mediators cumulates in lymphocyte death, preventing the host from mounting an effective adaptive immune response. excessive inflammation, coagulopathy and diffuse organ failure precedes death of the host. (**B**) In contrast, rVSV-EBOV-GP vaccination induces a regulated antiviral interferon response in the absence of the cytokine storm seen in EBOV infection. Effective activation and mobilization of antigen presenting cells (APCs) enables a robust.

The host response to rVSV-EBOV-GP vaccination has been primarily analyzed in nonhuman primates at the functional and transcriptional levels [3,56,61,97,98,99,100] (Figure 1, Table 3). Intramuscular vaccination with 10 million PFU of rVSV-EBOV-GP results in complete protection and no detectable viremia [56,61]. Although a large increase in the number of proliferating central and effector memory CD4 and CD8 T cells was noted 7–14 DPV, the frequencies of interferon (IFN)-γ-secreting EBOV-GP-specific T cells were low [61,101]. Increases in the frequencies of proliferating marginal-zone, antibody-producing B cells and memory B cells are also noted at 14–21 DPV, correlating with the increased levels of neutralizing and non-neutralizing EBOV-GP-specific IgG [61].

Transcriptional analysis of NHP whole blood and peripheral blood mononuclear cells (PBMCs) post-vaccination reveals gene expression changes in PBMC that peak 7 DPV and resolve 14 DPV, at the time when frequencies of proliferating T and B cells peak [98,99]. Transcriptional responses in whole blood are detected as early as 3 DPV and are also resolved at 14 DPV [98,99]. Notably, the magnitude of the transcriptional response in PBMC (~60 differentially expressed genes, DEGs) is notably lower than that in whole blood (~500 DEGs) at 7 DPV, suggesting roles for additional immune and non-immune cells that are lost upon gradient centrifugation (Table 3). In both PBMC and whole blood, large increases in the expression of interferon stimulated genes (ISGs) and genes involved in innate antiviral immunity (e.g., *IFIT2, OAS2, IFI44L, GBP6*) are detected at 3 and 7 DPV, while upregulation of DEGs involved in humoral immunity (e.g., *JCHAIN, BAFF, LYN*) are only detected when analyzing whole blood (Table 3) [98,99]. This correlates well with the later appearance of EBOV-GP-specific antibodies. Transcriptional responses in whole blood at 7 DPV are also characterized by the upregulation of DEGs with roles in antigen presentation (e.g., *PSMB9, HLA-DQB1 TAP2*), cell signaling pathways (e.g., *TNFSF10, IRF2, STAT5*), inflammation (e.g., *NFKB1A, IL-1B*) and T cell immunity (e.g., *TAGAP*), which correlates with increased levels of memory T cell [3,97,98,99]. The main contributors of these transcriptional changes are antigen presenting cells, NK cells and B cells, as well as non-immune cells given the discrepancy in transcriptional responses seen in PBMC and whole blood [98,99].

Limited transcriptional data are available for the human response to rVSV-EBOV-GP exists [100,102]. Analysis of the host transcriptional responses at 1, 3 and 7 DPV reveals a large induction of DEGs at 1 DPV (10,123) that declines at 3 DPV (3478) and 7 DPV (268). The most notably upregulated DEGs at 1 DPV include genes that play a role in chemotaxis (e.g., *CCL8, CCL2*), NK cell immunity (e.g., *CXCL10*) and, as reported in NHP, antiviral defense (e.g., *ISG15, IFIT1, HERC5*) (Table 3). Approximately 15 DEGs (e.g., *TIFA, TRAF2, NFKB, TNFRSF1A*) within to the CXCL10/IP10 pathway were found to correlate with EBOV-GP-specific antibody responses. Additionally, protein levels of key cytokines, such as IL-6 and IFN-alpha, and chemokines like MCP-1 and CXCL10/IP10 are elevated early following vaccination [100]. As described in NHP samples, an increase in the number of CXCR6+ NK cells and antigen-presenting cells (APC, monocytes and dendritic cells) is detected post vaccination as early as 1 DPV. Furthermore, a correlation between NK cells and EBOV-GP-specific antibody response was observed, suggesting that NK cell memory may be associated with and play a role in vaccine-mediated protection [103]. Additional in-depth immune cell phenotyping and cell-specific transcriptional studies should be carried out in humans to provide a more comprehensive understanding of the immune response to rVSV-EBOV.

## 6. Correlates of rVSV-mediated Protection

Early studies using NHP models demonstrated that antibodies and CD4 T cells are necessary for rVSV-EBOV-mediated protection against lethal infection while CD8 T cells play a minor role [61,66]. Specifically, all animals depleted of CD4 T cells during vaccination succumbed to EBOV challenge while those depleted of CD4 T cells only during EBOV challenge survived. Animals depleted of CD4 T cells during vaccination also failed to generate EBOV-GP-specific IgG antibodies, Collectively, these data indicate that CD4 T cells play a critical role by providing help to B cells rather than as effector cells in rVSV-EBOV-GP-mediated protection. Vaccinated NHP survivors exhibit high levels of EBOV-GP-specific IgG prior to challenge and increased levels of proliferating T and B cells, while those who succumb to infection do not [35,56,60,61,65,104]. This correlates with observations that human survivors of EBOV infections often have greater levels of EBOV-specific IgG and IgM compared to fatal cases during infection and in subsequent years post recovery, although data are limited due to a lack of samples [6,105,106,107]. Furthermore, immunocompromised macaques infected with simian human immunodeficiency virus (SHIV) and possessing low levels of CD4 T cells generate poor EBOV-GP-specific antibodies responses following vaccination, and, consequently, succumb to challenge with EBOV [91]. Decline of CD4 T cells following infection is also associated with fatal EVD in humans [108,109]. These findings suggest a critical integration of CD4 T cell responses and B cell-mediated immunity, which is further supported by the observation that CD4 T follicular helper (Tfh) cells are significantly induced in human rVSV-EBOV-GP vaccinees and their frequency correlates with levels of cytokines associated with B cell responses (e.g., IL-4, IL-6 TNF-alpha, IL-12) [110]. Tfh cells are the limiting factor for germinal center- and humoral immunity and have been shown to be critical for the development of humoral immunity in multiple virus infections and vaccinations, including influenza virus and yellow fever virus [110,111,112,113,114,115,116,117,118,119,120,121,122,123,124,125,126].

The role of humoral immunity in rVSV-EBOV-mediated protection is exemplified by the success of several monoclonal antibody (mAb) therapies. To date, all FDA-approved and in-trial mAb antibody therapies target EBOV-GP. Zmapp, the first mAb antibody cocktail composed of three mAbs, is safe and improves overall mortality rates in humans, as seen during the West Africa outbreak of 2013–2016 [107,108,109]. Zmapp also shows complete protection in NHPs that receive the cocktail 24 h after exposure and partial protection in those that receive it 48 h after exposure to a lethal dose of EBOV Kikwit, although these results vary across studies [127,128,129]. Improved mAb formulations (e.g., REGN-03, MIL77E), which target EBOV-GP more efficiently demonstrated greater effectiveness than Zmapp in clinical trials and preliminary animal models, including a single mAb formulation (mAb114) derived from a survivor of the EBOV Kikwit strain 11 years post-recovery that protects NHPs as late as 5 day post EBOV infection [130,131,132,133,134,135]. It must be noted that mAb therapy-mediated protection is incomplete but can synergize with rVSV-EBOV-GP vaccination given as late as a day prior to infection in NHPs to confer complete protection [58,136,137]. In contrast, convalescent plasma, composed of multiple EBOV-GP-specific antibodies, shows very limited efficacy [34,138,139,140].

Although CD8 T cell are not necessary, they do play a role in rVSV-EBOV-GP-mediated protection [61]. As noted, rVSV-EBOV-GP vaccination is characterized by significantly increased levels of proliferating CD8 effector and central memory T cells in addition to proliferating memory B cells in NHPs [61]. In humans vaccinated with a low (3 × 10^5^ PFU), intermediate (3 × 10^6^ PFU) or high (2 × 10^7^ PFU) dose of rVSV-EBOV-GP (NCT02283099), overall T cell responses increase from 0 to 56 DPV while cytotoxic CD8 T cell responses increase only in the high dose group [141]. Additionally, NHPs depleted of CD4 T cells during vaccination prior to challenge with EBOV exhibit an extended time to death compared to unvaccinated counterparts, suggesting a contribution of cellular adaptive immunity to rVSV-EBOV-mediated protection. EVD human survivors also exhibit proliferation of activated proliferating CD8 T cells [142,143]. Interestingly, most CD8 T cells in unvaccinated EVD patients are reactive towards EBOV nucleoprotein (NP) [142,143]. Whether these NP-specific CD8 T cells are needed for protection—and the definitive role of cellular adaptive immunity in vaccine-mediated protection—remain to be determined.

## 7. Host Transcriptional Response to Ebola Virus Infection With or Without Vaccination

The transcriptional and physiological responses to EVD in NHPs and humans are reviewed extensively elsewhere [142], but a brief description of the transcriptional response is provided here to provide additional context to the host response to rVSV-EBOV-GP in NHPs (Figure 1A, Table 3) [2,144,145]. Fatal EBOV infection in NHPs is associated with substantial gene expression changes that correlate with viremia and immune dysregulation. Innate antiviral defense genes, namely ISGs (e.g., *MX1, OAS1, STAT1, STAT2, ISG15*), are upregulated early in fatal infection and their expression increases dramatically throughout infection indicative of dysregulated innate immune response [76,77,108,144,146,147,148,149,150]. This is followed by increased expression of pro-inflammatory cytokines (e.g., *IL6, IL1B, IL24, CCL2*) and chemokines (e.g., *CXCL10, MCP-1, CXCL13*), which is consistent with the cytokine storm characteristic of EVD [76,77,108,144,146,147,148,149,150]. An increase in apoptotic gene expression (e.g., *FAS, BCL2A1, CASP3*) and concomitant decrease in lymphocytes and lymphocyte cell activation markers (e.g., *BTK, LTA, CD274, CD3, CD38*) later in infection in line with the lymphopenia and the lack of an adaptive immune response observed in EVD [76,109]. The loss of lymphocytes is believed to be due to the combination of the cytokine storm and failure to activate and mobilize APCs like dendritic cells and macrophages [76,77,146,147,148,151,152,153,154]. Additionally, transcriptional analysis of monocytes isolated ex vivo from cynomolgus macaques infected with EBOV shows reduced expression of genes critical for antigen processing and presentation [99]. Changes in the expression levels of genes regulating coagulation and blood vessel development (e.g., *PTFAR, THBS1, MMP9*) correlate with the development of hemorrhage and coagulopathy [3,76,77,144]. The upregulation of neutrophil-associated genes (e.g., *ITGAM, MMP25, IL8, NCF1*) in parallel with granulocytosis and neutrophilia further supports a role for neutrophils in EVD pathology [3,76,77]. Indeed, the major source of transcriptional changes originates from infected monocytes and neutrophils [77,155]. These results are paralleled in human studies comparing nonfatal and fatal infections, with additional dysregulation seen in lipid metabolism, amino acid homeostasis and inflammatory mediators [108,146].

Recent studies have examined the transcriptional response in whole blood following challenge in animals vaccinated as early as 4 weeks and as late as 3 days before challenge [98,99]. These studies demonstrate that the transcriptional profile of NHPs vaccinated with rVSV-EBOV-GP before EBOV challenge is distinct from that seen in naïve animals challenged with EBOV (Figure 1B) [3,97,98,99]. Very few differentially expressed genes (DEGs) (<350) are detected in NHPs challenged with EBOV 4 weeks after vaccination compared to unvaccinated NHPs and indicate upregulation of ISGs and downregulation of inflammatory, metabolic, stress-related and cell cycle-related genes [76,77,98,99]. In contrast, a substantial number of DEGs is detected 2-4 weeks after EBOV challenge in macaques vaccinated 7 days before vaccination (>1400 DEGs), consistent with potentially a boosting of the adaptive immune response [98]. Specifically, genes involved in metabolism and adaptive immunity are upregulated at 14 and 28 days post-challenge (DPC), including those related to T and B cell activation (e.g., *CD28, LAT, MEF2C, IGHA1, SLAMF1*). Downregulated genes largely suggest suppression of myeloid cell activation and inflammation (e.g., *TLR4, CXCR1, TNFAIP6, CD55*).

NHPs depleted of CD4 T cells during vaccination (resulting in the lack of antibodies) have a similar transcriptional profile following EBOV challenge as that detected in unvaccinated animals [99]. DEGs involved in innate immune defense, inflammation and stress (e.g., *SERPING1, IL1RN, IFIT5*) are significantly upregulated at 4 and 7 DPC. Interestingly, in contrast to unvaccinated animals, DEGs with roles in adaptive immunity are not downregulated in macaques depleted of CD4 T cells during vaccination, suggesting that the presence of CD8 T cells may alter disease trajectory in these animals. However, by 7 DPC expression of genes related to coagulation is dysregulated in CD4-depleted animals consistent with EVD. Transcriptional analysis of PBMC obtained from vaccinated NHPs that were CD8-depleted during vaccination and challenged with EBOV Kikwit demonstrate a large induction of DEGs. These DEGs enrich to inflammation and coagulation despite the fact that these animals are show no viremia or clinical sign of EVD, suggesting a low level of viral replication in tissue reservoirs such as lymph nodes [61,98]. Additional studies focused on CD8 T cell will need to be performed to confirm the exact contributions of CD8 T cells to both vaccine-mediated protection and survival following challenge.

## 8. rVSV-EBOV-GP and Rapid Protection: the Role of Innate Immunity

**Figure 2:**
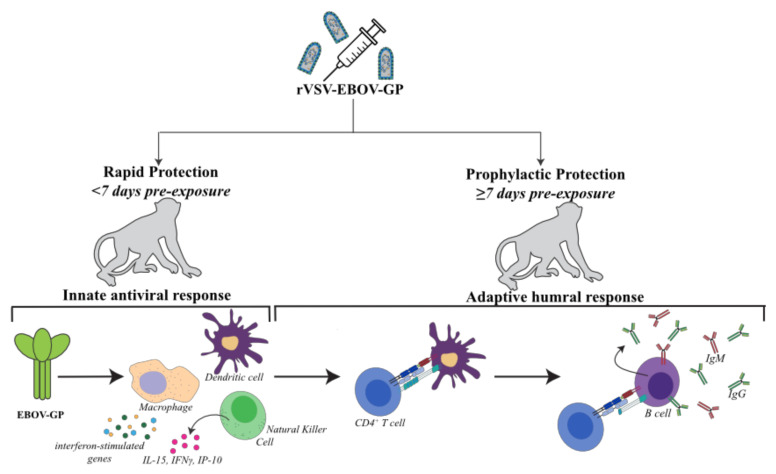
rVSV-EBOV-GP mechanisms of protection in nonhuman primates. rVSV-EBOV-GP vaccination <7 days before EBOV challenge in nonhuman primates induces a protective immune response predominantly driven by innate immune and characterized by activated antigen presenting cells (e.g., macrophages, dendritic cells), NK cells secreting cytokines like IL-15 and the upregulation of antiviral interferon-stimulated genes in response to EBOV-GP recognition. In contrast, protection in macaques vaccinated >7 before EBOV challenge is mediated by humoral adaptive immunity, in which APC-stimulated CD4+ T cells promote B cell maturation and the production of IgG and IgM antibodies targeting EBOV-GP.

In addition to the role of humoral immunity, there is a clear role for innate immunity in rVSV-EBOV-mediated rapid protection (defined as protection conferred when vaccination occurs <7 days before challenge) (Figure 2). Partial protection is conferred to macaques vaccinated with rVSV-EBOV-GP as late as 1–3 days before EBOV challenge before the appearance of GP-specific antibodies. Interestingly, partial protection (67%) was also reported in NHPs that were vaccinated twice with rVSV-MARV-GP (which shares <70% nucleotide identity with EVOV-GP), at 1 h and 24 h after EBOV [56,79,98]. These data suggest a critical role for innate immune responses engendered by rVSV in the mediating early protection.

Rapid protection conferred by rVSV-EBOV-GP when administered 3 days before EBOV challenge is associated with a remarkably different transcriptional response reflective of disease outcome [56,98]. The survivors upregulate expression of genes that play an important role in development of humoral immune genes, as well as those involved in cell migration, inflammation, innate antiviral immunity, and cellular cytotoxicity [98]. In contrast, non-survivors exhibit similar transcriptional signatures as those detected in non-vaccinated animals [76,77,144,146,147,148,149,150]. These observations suggest that rapid protection may be mediated by innate immunity at early time points after challenge in parallel with the appearance of innate cytokine signatures including type-I IFN and IL-15 [56,61]. A robust innate immune response may keep viral replication to a minimum “buying time” until the adaptive immune response can develop approximately a week after vaccination. The robust innate antiviral transcriptional responses seen 3 days post-vaccination may indicate a contribution from the VSV vector, which transiently replicates at low levels in vaccinees [78,79,156,157]. This also provides rationale for the partial protection afforded by 2 doses of rVSV-MARV-GP to EBOV-challenged macaques, although the converse-partial protection against MARV by rVSV-EBOV-has not been observed [79,98].

Rapid antiviral innate immunity may also be mediated by EBOV-GP, which has been shown to induce activation of human macrophages and DCs via TLR4 signaling [158,159,160,161]. This effect of membrane-bound GP (predominant form during vaccination) is distinct from the effects of soluble GP (sGP), which is produced in large quantities during EBOV infection: for instance, in vitro stimulation of DCs and macrophages with sGP thwarts migratory ability and induces a robust cytokine response while simultaneously increasing vascular permeability [4,162,163]. Whereas TLR4 stimulation has been shown to activate DCs and macrophages to differentiate and macrophages to polarize into an anti-inflammatory state [6,109,158,161,164,165,166,167]. Studies in mice demonstrate that vaccination with three doses of purified EBOV-GP confers partial protection (70% survival) from lethal EBOV infection in [157]. This protection is abrogated by TLR4 inhibition [151,152,153,154].

NK cell-mediated innate immunity has been implicated, with less compelling evidence, in rVSV-EBOV-mediated protection. Frequency of NK cells is elevated in vaccinated NHPs following vaccination and challenge with a signature of NK cell-associated cytokines such as IL-15 and IFN-gamma appearing early post-vaccination when T and B cell immunity are lacking [56,78]. NK cells may also be necessary for vaccine-mediated protection since NK cell-depleted mice vaccinated with virus like particles (VLPs) bearing purified EBOV VP40 and GP proteins succumb to infection [168]. The role of NK cells in rVSV-EBOV-mediated protection is still tentative, but given that NK cells are significantly increased in survivors, induction of NK cells may have a significant role in the early innate immune response to EBOV when vaccination occurs shortly before or after EBOV exposure [63,78,108,168,169]. The correlation between humoral immunity and NK cells in human vaccination studies also suggests a role for NK cells [100]. However, the role of NK cells in individuals exposed a greater time after vaccination (>7 days), may be minimal if NK cell and adaptive immunity are not inherently integrated as other innate immune mechanisms, including TLR4 signaling may be.

## 9. rVSV-EBOV-GP Versus Attenuated Ebola Virus Vaccines

**Figure 3:**
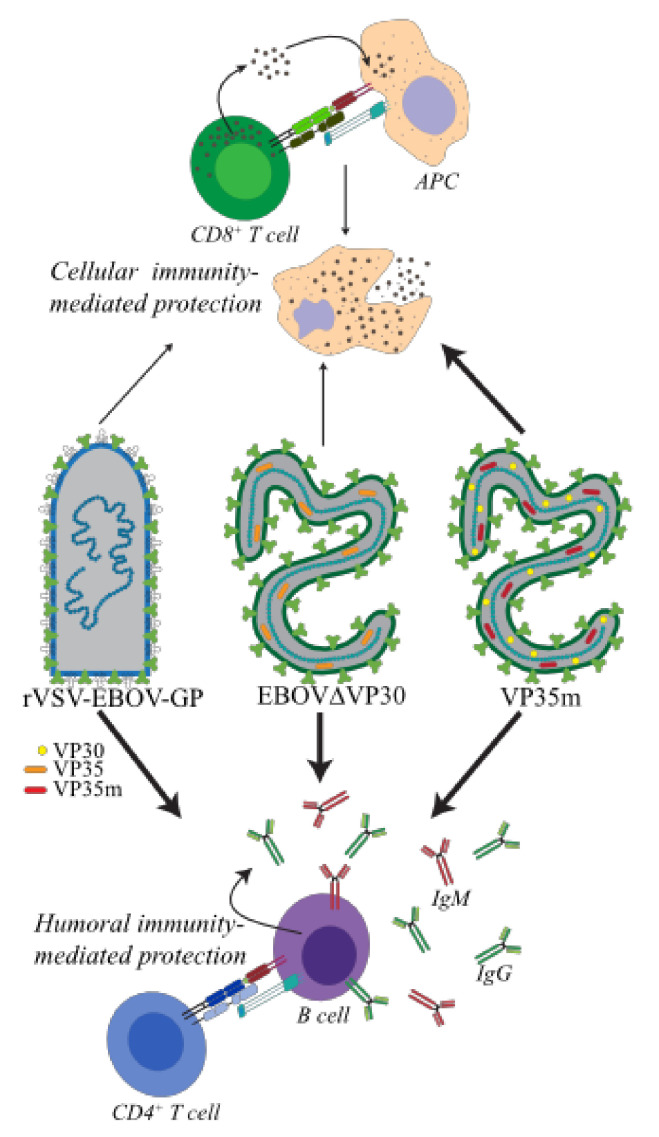
Mechanisms of protection of rVSV-EBOV-GP in comparison to whole-virus candidate EBOV vaccines.

VP35 and VP30 are Ebola virus proteins critical for the viral life cycle and host immune antagonism [154,170,171,172,173,174,175,176,177,178]. Recent studies have demonstrated that mutations in VP35 or deletion of VP30 from the EBOV genome (VP35m and EBOVΔVP30, respectively) attenuates EBOV pathogenicity, and confers protective immunity to subsequent challenge with wild type EBOV. In both cases, VP35m and EBOVΔVP30 function as whole-viruses containing a myriad of immunogenic antigens to the host as opposed to rVSV-EBOV-GP, in which only the single EBOV glycoprotein is presented. The mechanisms of protection of these attenuated/replication-deficient EBOV share some properties with those conferred by rVSV-EBOV-GP (Figure 3).

VP35 is a key replication cofactor with an additional role in antagonizing the host innate antiviral immune response through inhibition of the type I IFN signaling pathway and interfering with dendritic cell maturation [154,170,171,172,173,174,175,176]. Viral double-stranded RNA is detected by RIG-1 like receptors (RLRs) RIG-1 and MDA5, which leads to the phosphorylation, activation and translocation of IRF3 to the nucleus to induce transcription of type I IFN and ISGs [179,180]. VP35 dysregulates this antiviral response by binding dsRNA to prevent IRF3 phosphorylation, ultimately inhibiting RLR-mediated expression of IFN and ISGs [154,172,173,174,175,176]. Administration of VP35m that contains three point mutations in VP35 that reduce dsRNA-binding ability results in minor to no symptoms and the induction of both innate and adaptive responses in cynomolgus macaques, including increased levels of proliferating lymphocytes and virus-specific IgG [79,181,182,183,184]. Furthermore, cynomolgus macaques infected with VP35m exhibit few to no signs of disease and are fully protected from a lethal dose of EBOV Kikwit in a dose dependent manner [79,181,182,183,184].

Humoral immunity is believed to be the major mechanism of protection provided by prophylactic rVSV-EBOV-GP vaccination while cellular immunity plays a smaller role. Vaccination with VP35m induces notable T and B cell responses. Protection mediated by the replication incompetent EBOV ΔVP30 likely provokes mainly humoral responses.

Comparative transcriptional analysis of VP35m and rVSV-EBOV-GP reveals that administration of either rVSV-EBOV-GP or VP35m induces robust changes in ISGs and cytokine signaling pathways as early as 3 days post administration. However, only VP35m induces DEGs related to myeloid cell immunity, inflammation, and T cell signaling as early as 3 days post infection (dpi) [98,99,149]. Evidence of myeloid cell immunity and the adaptive immune response induced by rVSV-EBOV-GP is not seen until 7 DPV. The host transcriptional changes in response to rVSV-EBOV-GP are also consistently smaller in magnitude compared to those observed following VP35m infection. Furthermore, the host transcriptional response to VP35m infection persists until 10 dpi where changes in adaptive immunity are evident while the response to rVSV-EBOV-GP is resolved by then [149]. Flow cytometry data collected following vaccination indicate that both VP35m infection and rVSV-EBOV-GP vaccination both induce increases in the frequencies of proliferating CD4 and CD8 memory subsets and B cells, with proliferative bursts in VP35m infection occurring earlier (3–14 dpi) than that after vaccination (~14 DPV) [98,99,149]. VP35m also activates innate immune cells, as evidenced by enhanced frequencies of activated monocytes and dendritic cells, although whether this occurs with rVSV-EBOV-GP vaccination has not been investigated. These findings support the idea that VP35m may contains additional immunogenic properties compared to rVSV-EBOV-GP (Figure 3).

VP30 plays an essential role in viral replication and regulation and initiation of viral transcription [177,178]. Deletion of VP30 from the Ebola virus genome (EBOVΔVP30) results in a replication-incompetent virus that is nonpathogenic in animals, including mice, guinea pigs and nonhuman primates [39,185,186,187]. Vaccination of nonhuman primates with a single dose (1 × 10^7^ PFU) or two doses of EBOVΔVP30 fully protects animals from challenge with a lethal dose of EBOV Kikwit [187]. However, several animals receiving a single dose experienced fever and/or viremia following challenge. Vaccination is characterized by dose-dependent increase in GP-, NP- and VP40-specific IgG, and neutralizing anti-GP antibodies. The number of IFN-gamma secreting mononuclear cells also increases in a dose-dependent manner, suggesting a possible cellular response in addition to a humoral one (Figure 3). Additional studies will need to be performed to determine the correlates of protection associated with either VP35m- or EBOVΔVP30-mediated protection and the advantage (or disadvantage) of including multiple EBOV antigens beyond EBOV-GP.

## 10. Conclusions

rVSV-EBOV-GP, a safe and efficacious vaccine, represents a critical advancement in the management of Ebola virus disease and provides a novel platform for the design of additional vaccines targeting emerging pathogens. Curiously, rVSV-EBOV-GP is effective both prophylactically and post-exposure to EBOV in NHP models, although mechanisms of protection are inconclusive, but with most compelling data indicating a central role for humoral immunity complemented and enabled by innate immunity. Early innate virus- and virus-non-specific responses may provide a controlled inflammatory and antiviral defense response that leads to the activation of antigen presenting cells, which in turn, activates CD4 T cells that modulate B cell activation. Strong relations between EBOV-GP-specific antibody titers and the dispensable role of CD8 T cells collectively support this model. Despite the current findings, additional studies are needed to fill key gaps in our knowledge of rVSV-EBOV-mediated protection and elucidate the powerful protection afforded by this novel vaccine platform. Unanswered questions include investigating the dynamics of Tfh, CD8 T cells and NK cell activation after vaccination and challenge; the mechanisms conferring synergy between innate and adaptive immune response to rVSV-EBOV-GP; the durability of single dose vaccination; and determination of key signatures of successful vaccination to create powerful predictive models and facilitate both the design and development of effective antivirals and therapeutics. An approach integrating clinical, transcriptomic, metabolomic, proteomic and immunological techniques to analyze both preclinical and clinical responses to rVSV-EBOV-GP vaccination and subsequent exposure to Ebola virus will be critical in completing our understanding of rVSV-EBOV-mediated protection and validating a robust vaccine platform for the development of other viral vaccines.

## Figures and Tables

**Table 1:** Outbreaks and Identification of Ebola viruses.

Year	Country	Species	# Reported Cases	Case Fatality Rate (%)
2019–Present	DRC *	Zaire	ongoing	NA
2018–2020	DRC, Uganda	Zaire	3,524	66
2018	DRC	Zaire	54	61
2017	DRC	Zaire	8	50
2014	DRC	Zaire	66	74
2016	Sierra Leone	Bombali	#	#
2013-2016	Guinea, Sierra Leone, Liberia	Zaire (Makona) *	28652	40
2012	Uganda	Sudan	6	50
2012	Uganda	Sudan	11	36
2011	Uganda	Sudan	1	100
2008	DRC	Zaire	32	47
2007	Uganda	Bundibugyo	149	25
2007	DRC	Zaire	264	71
2004	South Sudan	Sudan	17	41
2003	RC *	Zaire	35	83
2002	RC	Zaire	143	90
2001	RC	Zaire	57	75
2001	Gabon	Zaire	65	82
2000	Uganda	Sudan	425	53
1996	South Africa	Zaire	2	50
1996	Gabon	Zaire	60	75
1996	Gabon	Zaire	37	57
1995	DRC	Zaire (Kikwit)	315	79
1994	DRC	Tai Forest	1	0
1994	Gabon	Zaire	52	60
1989	USA	Reston	#	#
1979	Sudan	Sudan	34	65
1977	DRC	Zaire	1	100
1976	Sudan	Sudan	284	53
1976	DRC	Zaire (Mayinga)	318	88

* DRC Democratic Repulication of Congo; * RC Republic of Congo; * () denotes emergence of a Zaire strain; # not detected in humans and/or does not cause disease.

**Table 2:** rVSV-EBOV-GP clinical trials.

Clinical Trial ID	Phase	Trial Classification	Start	End	*n* (patients)	Location(s)
NCT02269423	I	Double-blind, randomized placebo-controlled dose escalation	October 2014	August 2015	39	USA
NCT02280408	I	Double-blind, randomized placebo-controlled dose escalation	October 2014	December 2015	39	USA
NCT02283099	I	Open-label dose escalation	November 2014	November 2015	30	Germany
NCT02287480	I/II	Double-blind, randomized placebo-controlled dose finding	November 2014	November 2016	115	Switzerland
NCT02374385	I	Double-blind, randomized placebo-controlled dose ranging	November 2014	June 2015	40	Canada
PACTR201411000919191	I	Open-label dose escalation	November 2014	July 2020	115	Switzerland Gabon Kenya
NCT02314923	Ib	Double-blind, randomized placebo-controlled dose ranging	December 2014	June 2016	513	USA
NCT02296983	I	Open-label dose escalation	December 2014	September 2016	40	Kenya
NCT02933931	I	Observational cohort	November 2016	April 2020	100	Switzerland
NCT02344407	II	Double-blind, randomized placebo-controlled	January 2015	June 2020	1,500	USA
NCT02876328	II	Randomized, placebo-controlled	March 2017	January 2021	4,789	USA France England Liberia
NCT03031912	II	Double-blind, randomized placebo-controlled	August 2017	December 2019	200	USA
PACTR2015030011057193	III	Open-label, cluster-randomized ring vaccination	April 2015	July 2015	7,651	Guinea
NCT02378753PACTR201502001027220	II/III	Open-label, individually randomized, controlled	April 2015	November 2016	8,651	Sierra Leone
NCT02503202	III	Randomized, placebo-controlled dose evaluation	August 2015	May 2016	1,197	USA Canada Spain

**Table 3:** Representative differentially expressed genes (DEGs) detected in response to EBOV infection or rVSV-EBOV-GP vaccination.

Category	EBOV Infection Nonhuman Primate DEGs	EBOV Infection Human DEGs *	rVSV-EBOV-GP Vaccination Nonhuman Primate DEGs	rVSV-EBOV-GP Vaccination Human DEGs **
**Antiviral**	*STAT1, STAT2, MX1, OAS1, IFIT5, IFIH1, DDX60, ISG15, IFIT1, HERC5, RSAD2*	*IFI27, OASL, OAS3, MX1, OAS2, RSAD2, IFIT1, IFIT2, IFIT3, IFITM1*	*STAT1, STAT2, MX1, OAS1, IFIT5, IFIH1, DDX60, ISG15, IFIT1, HERC5, RSAD2, IFI44L*	*USP18, ISG15, IFIT1, HERC5, RSAD2*
**Inflammatory**	*IL18RAP, ILI1B, IL6, TLR4, RELA, TNFalpha, JUN, S100A8, HBB, NFKB*	*TNFRSF10C, ILI1R2*	*FCER1A, S100A9, C1RL, MYD88, TNFAIP6, MMP9, TLR4, IL1B, NFBIA*	*TIFA, HK1, NFKB1, NFKB2*
**Apoptotic, Cell Death**	*BCL2A1, BCL2, BAK1, CASP1, CASP5, FAS, TNFSF10, FAIM3, PYCARD*	*BCL2A1, XAF1, NAIP, CARD6, CLFAR, MKLKL, CASP7, AIM3*	*BAK1, FAST, TNFRSF10A, IFI27*	*MKRN1*
**Chemotactic**	*CXCR1, CXCR5, CCR7, IL1RA, MIP1alpha, CCL2, CCL8, IP10*	*CXCR1, CXCR2*	*CXCR1, CXCR2*	*CCL2, CCL8, IP10*
**Leukocyte-mediated** ***Innate***	*CD44, CD40LG, CYBB, SELP, IL8, KLF6, DOCK8, PSEN1*	*A1P9, MMP25, FPR1, FPR2, CSF3R, FCGR3B, CEACA8, ITGAM, LRG1, MPO*	*FPR2, GCA, CXCR, CXCL8*	*LAMP3*
**Leukocyte-mediated** ***Adaptive***	*CD274, ZAP70, CD3E, CD8A, B2M, CD40, BL6, BTK, SYK, LYN, PTPRC*	*CD4, CD247, LAT, ZAP70, HLA-A*	*GZMA, IL-27, CD83, CD1C, TCF7, CD2, CD4, CD28, ZAP70, CD19, IGHA1, SLAMF1, LYN*	

* See Eisfeld et.al. (2017), reference 146; ** See Rechtien et. al. (2017), reference 100.
